# *Coffea arabica*: An Emerging Active Ingredient in Dermato-Cosmetic Applications

**DOI:** 10.3390/ph18020171

**Published:** 2025-01-27

**Authors:** Grațiana Ruse, Alex-Robert Jîjie, Elena-Alina Moacă, Dalia Pătrașcu, Florina Ardelean, Alina-Arabela Jojic, Simona Ardelean, Diana-Simona Tchiakpe-Antal

**Affiliations:** 1 Discipline of Pharmaceutical Botany, Faculty of Pharmacy, “Victor Babes” University of Medicine and Pharmacy Timisoara, 2nd Eftimie Murgu Square, 300041 Timisoara, Romania; gratiana.ruse@umft.ro (G.R.); ardelean.florina@umft.ro (F.A.); alina.jojic@umft.ro (A.-A.J.); diana.antal@umft.ro (D.-S.T.-A.); 2 Department of Toxicology, Drug Industry, Management and Legislation, Faculty of Pharmacy, “Victor Babes” University of Medicine and Pharmacy Timisoara, 2nd Eftimie Murgu Square, 300041 Timisoara, Romania; alex-robert.jijie@umft.ro (A.-R.J.); alina.moaca@umft.ro (E.-A.M.); patrascu.dalia@umft.ro (D.P.); 3 Research Centre for Pharmaco-Toxicological Evaluation, Faculty of Pharmacy, “Victor Babes” University of Medicine and Pharmacy Timisoara, 2nd Eftimie Murgu Square, 300041 Timisoara, Romania; 4 Faculty of Pharmacy, Vasile Goldis Western University of Arad, Revolutiei Bvd 94, 310130 Arad, Romania

**Keywords:** *Coffea arabica*, leaves, seeds, dermato-cosmetology, chlorogenic acids, caffeine, skin health

## Abstract

**Background**: *Coffea arabica*, commonly known as Arabica coffee, has garnered attention in recent years for its potential applications in dermato-cosmetic formulations. This review aims to critically evaluate the emerging role of *Coffea arabica* as an active ingredient in skin care products, focusing on its bioactive compounds derived from both the leaves and beans, mechanisms of action, and efficacy in dermatological applications. A comparative analysis between the bioactive profiles of the leaves and beans is also presented to elucidate their respective contributions to dermato-cosmetic efficacy. **Results**: This review synthesizes findings from various studies that highlight the presence of key bioactive compounds in *Coffea arabica*, including caffeine, chlorogenic acids, and flavonoids. Notably, the leaves exhibit a higher concentration of certain phenolic compounds compared to the beans, suggesting unique properties that may enhance skin health. These compounds have demonstrated significant anticellulite, anti-inflammatory, antioxidant, photoprotective, anti-aging, antibacterial, and moisturizing properties. **Discussion**: This article delves into the biochemical pathways through which bioactive compounds derived from both the leaves and beans of *Coffea arabica* exert their beneficial effects on skin and hair health. Furthermore, this review highlights the growing trend of incorporating natural ingredients in cosmetic formulations and the consumer demand for products with scientifically substantiated benefits. **Conclusions**: The findings of this review underscore the potential of *Coffea arabica* as a valuable active ingredient in dermato-cosmetic applications. Its multifaceted bioactivity suggests that it can contribute significantly to skin health and cosmetic efficacy. Future research should focus on clinical trials to further validate these benefits and explore optimal formulation strategies for enhanced delivery and stability in cosmetic products.

## 1. Introduction

The use of *Coffea* seeds to obtain well-known and appreciated beverages made this plant one of the most famous worldwide. Its importance is not limited to the preparation of coffee but also extends to various fields and involves pharmaceutical uses [1], exploitation of other parts of the plant [2], or recycling of by-products resulting from coffee production [3]. There is also growing interest in its dermato-cosmetic applications [4,5].

*Coffea* species are shrubs belonging to the Rubiaceae family, the fourth largest family of angiosperms, and the tribe Coffeeae [3,6]. The *Coffea* genus includes 124 species [7]. Their evergreen leaves are lanceolate, shortly petiolate, with a reticulate venation and a shiny dark-green surface when they reach maturity [8]. Flowers are white or pink [9]. The fruits (“coffee cherries”) are drupes, each one containing two elliptical seeds (“coffee beans”), presenting a longitudinal furrow on the flat side [10,11]. Although most *Coffea* species have red fruits, 3% of *Coffea arabica* can have yellow cherries when they are ripe [3].

Two *Coffea* species are the most economically important and used for coffee production. These are *Coffea arabica*, known as Arabica coffee, and *Coffea canephora*, known as Robusta coffee [12]. *Coffea arabica* L. is an allotetraploid, meaning it has four sets of chromosomes derived from two diploid ancestors, resulting from the spontaneous hybridization between *Coffea canephora* Pierre ex A. Froehner and *Coffea eugenioides* S. Moore [7,13,14]. This genetic complexity contributes to its unique biochemical composition, which includes a variety of bioactive compounds, such as caffeine, phenolic derivatives, diterpenes, and other metabolites [15,16]. *Coffea arabica* represents about 63% of international trade, while *Coffea canephora* is 37% [2]. Arabica coffee is characterized by its superior flavor profile, lower caffeine content compared to its counterpart *Coffea canephora* (Robusta), and its susceptibility to various diseases, which makes it a more delicate crop to cultivate [3,4]. Other species cultivated on a smaller scale are *Coffea liberica*, *Coffea racemosa*, and *Coffea stenophylla* [12].

Originating from the highland rainforests of Ethiopia, *Coffea arabica* is one of the two most widely cultivated coffee species, accounting for approximately 70% of the world’s coffee production [17,18]. Its seeds were transported to Yemen in the 8th century. Plant expansion continued in further countries, reaching Europe at the beginning of the 17th century and later leading to its extensive cultivation in the intertropical regions [10]. Considering the increased worldwide consumption of coffee (a 4.2% increase in consumption was reported in coffee year 2021/2022 [19]), its cultivation presents a special interest. Among the biggest coffee producers are Brazil, Colombia, Indonesia, and Vietnam [3].

The first data on coffee were mentioned by Avicenna and refer to a beverage from Yemen called buncham used to treat stomach aches. Starting in the 12th century, coffee was increasingly known for its stimulant effects. Aqueous extracts from seeds have been used in Thailand for neurotonic and cardiotonic properties. Coffee coal was used in folk medicine in the treatment of stomatitis and festering wounds. Root sap was used in West India against scorpion bites, and the aphrodisiac effects of the roots were also known in Ethiopia [1]. Coffee husk is used to obtain the “cascara beverage”, which is consumed in Ethiopia and Yemen [20].

Coffee leaves possess a rich ethnomedicinal history in the regions where coffee cultivation is prevalent. In numerous countries, including Ethiopia, India, Indonesia, and Jamaica, sun-dried coffee leaves are utilized as a traditional substitute for tea, reflecting their cultural significance and versatility in local practices [21]. The consumption of coffee leaves extends beyond mere dietary use; they are employed by various communities for their purported medicinal properties [22,23]. In Ethiopia, infusions made from coffee leaves are traditionally administered as a laxative. In Mexico, coffee leaf infusion is utilized as a remedy against fever. Furthermore, in Nicaragua, decoctions of coffee leaves have been documented as effective herbal remedies for alleviating headaches and stomach pains [23,24]. In Peru, they are used to treat cough, while in Haiti, they are used for the treatment of asthenia, anemia, and edema [25].

Several stages exist between coffee fruit harvesting and obtaining the seeds ready to use for coffee preparation. After collection, the skin (exocarp) and the pulp (mesocarp) of the fruit are easily detached, and subsequent processes are required for the removal of other layers (mucilage—pectin layer, parchment—endocarp of the fruit, silverskin—integument). The seeds are then subjected to a drying process. The postharvest processing methods influence the quality of the coffee beverage [26].

The coffee plant provides several products, some of them with well-defined uses and others with potential uses still under exploration. Seeds are the most studied product of coffee plants. Green seeds do not have the pleasant aroma characteristic of roasted coffee beans. The odorous compounds are formed during the roasting process from non-volatile precursor molecules [26]. Despite this fact, they are appreciated as a source of antioxidants with multiple health benefits, and studies have shown positive effects on lipid profile, blood pressure, obesity, or insulin resistance [27]. Their interest in cosmetic applications is growing [28].

The silverskin represents the main by-product of coffee roasting [2]. It is the thin outer layer (integument) that covers raw seeds, and it is detached during roasting by exposure to high temperatures. It has been valued as a soil fertilizer and fuel, but its high content of fibers and antioxidant compounds suggests the possibility of use in the food sector (biscuits, bread, flakes, etc.) [29].

The cherry pulp is botanically the fleshy mesocarp of the fruit; it is removed in order to obtain the coffee beans. It contains chlorogenic acid and caffeine as major compounds and exhibits anti-aging and anti-hair-loss properties [28]. Processing coffee fruits involves drying as a first step and then the removal of the outer layers of the fruit (de-hulling). The residues produced in this process represent the coffee husks [30]. It may be used for the extraction of anthocyanins [31]. Another coffee by-product is the parchment, which represents the fruit endocarp, covering the seeds and separating them. It is a source of fiber with the potential to be added to foods, with benefits on blood glucose and serum lipid levels [32].

Spent coffee grounds are a waste product in coffee preparation, but, nevertheless, they are a source of phenolic compounds, lipids, and carbohydrates, with the potential to be re-used [33]. The coffee brewing process produces large amounts of spent coffee grounds that can be reused for the development of nutraceutical supplements or oil extraction [34].

Studies regarding coffee flowers are scarce. This part of the plant may be employed for the simultaneous coproduction of compounds such as caffeine, trigonelline, and melanoidins through pressurized hot water extraction [35].

**Figure 1:**
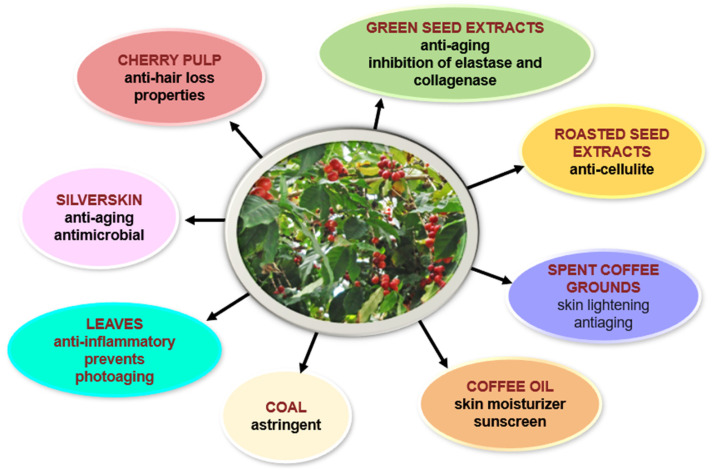
Main properties of dermato-cosmetical interest displayed by natural products derived from *Coffea arabica*.

In recent years, an impressive number of studies investigated and endorsed the vast potential of various natural products (leaves, green seeds, roasted seeds, oil, husk, silverskin) from *Coffea arabica* in dermato-cosmetology (Figure 1). Some of the most representative effects of leaf extracts include elastase and collagenase inhibition [28]; the prevention of photoaging [36]; and anti-inflammatory, antibacterial, and antioxidant effects [3]. Coffee silverskin has anti-aging and antimicrobial properties [37]. Spent coffee oil has been tested as a cleansing agent in makeup remover products, thus valorizing spent coffee as a promising renewable source for cosmetic formulations [38]. Coffee oil is appreciated in the cosmetic industry as an ingredient of sunscreen, moisturizer, or emollient products. It stimulates collagen synthesis and has antioxidant and anti-inflammatory potential. Topical green coffee oil formulations cause faster healing of wounds [39]. For coffee coal, resulting from the charring of roasted coffee seeds, an astringent effect was reported [1]. The versatility in cosmetic formulations is further enhanced by its pleasant aroma and sensory attributes, which can improve user experience and product appeal [40].

The objective of the present review is to provide a comprehensive overview of *Coffea arabica*, focusing on its chemical composition and effects relevant to dermato-cosmetology. By synthesizing the existing literature, we aim to elucidate the potential applications of *Coffea arabica* in skin care products and highlight the mechanisms through which its bioactive compounds exert beneficial effects on skin health. Given the huge number of articles addressing this subject, the selection of the most relevant studies was made using search term combinations, including the plant name (“*Coffea arabica*”), the main subject of the article (“skin”), with coffee product types (“ leaves”, “seeds”, “extract”), or phytochemicals (“alkaloids”, “caffeine”, “trigonelline”, “chlorogenic acid”, “flavonoids”, “phenolic”, “diterpenes”, “kahweol”, “cafestol”) or bioactivities (“anti-inflammatory”, “antioxidant”, “anti-aging”, “antibacterial”), or the term “cosmetic”. While some general information is given on the dermato-cosmetic interest in coffee by-products, this review focuses on the phytochemistry and dermato-cosmetic relevance of coffee leaves, green seeds, and roasted seeds. Articles published in the last ten years were privileged. Furthermore, this review explores the implications of scientific findings for the development of innovative cosmetic formulations that leverage the natural properties of *Coffea arabica*, ultimately contributing to the advancement of dermato-cosmetology as a field.

## 2. Chemical Composition of *Coffea arabica*

### 2.1. Phytochemicals in Coffea arabica Leaves

*Coffea arabica* leaves are rich in various bioactive compounds that contribute to their health benefits and applications in dermato-cosmetic formulations. These phytochemicals include a diverse range of compounds, such as alkaloids, polyphenols, diterpenes, and other compounds [41,42]. Among alkaloids, coffee leaves contain purine derivatives (caffeine, theophylline, and theobromine) as well as the pyridine alkaloid trigonelline, with caffeine and trigonelline being the most prominent. Studies have reported caffeine concentrations of about 15 mg/g in young leaves and 6 mg/g in mature leaves of *Coffea arabica* species [24]. This suggests that young leaves are richer in caffeine, which may be relevant for their use in food supplements and dermato-cosmetic products. On the other hand, the variability of caffeine content in *Coffea canephora* leaves is higher, with reported concentrations ranging from 2.8 to 7.7 mg/g, in contrast to the more constant levels observed in mature *Coffea arabica* leaves, which range from 2.5 to 6.0 mg/g. This variability can be attributed to factors such as genetic differences between different genotypes and the influence of climatic conditions [24,43]. The catabolic pathway of caffeine in plants is complex, involving several steps of methylation and degradation, in which caffeine is first converted to theophylline and subsequently to 3-methylxanthine and xanthine [43]. The rate-limiting step in this catabolic process is often the conversion of caffeine to theophylline, which is significantly slower than the degradation of theophylline itself [44]. In addition, the findings of Ashihara et al. suggest that, although caffeine catabolism is a well-established process in *Coffea arabica*, it is often hindered at the theophylline conversion step, leading to an accumulation of caffeine in young leaves [43]. This phenomenon emphasizes the complicated balance between caffeine biosynthesis and degradation, which is influenced by both genetic and environmental factors. Trigonelline is the second most prevalent alkaloid found in coffee leaves and is recognized for its potential role as a precursor in nicotinamide adenine dinucleotide (NAD) biosynthesis. However, this function appears to be limited to the early stages of germination in certain plant tissues [24]. This compound has important roles in plant metabolism and is considered a reservoir for NAD biosynthesis. The concentration of trigonelline in *Coffea arabica* leaves is about 12 mg/g in young leaves and 6 mg/g in mature leaves, suggesting its higher utilization in the early stages of leaf development [24]. Theobromine concentration in *Coffea arabica* leaves is low, about 0.8 mg/g in young leaves and 0.04 mg/g in mature leaves [24]. This indicates a significant decrease in theobromine as the leaves age.

One of the most important classes of phytochemicals found in *Coffea arabica* is polyphenols, including phenolic acids, flavonoids, xanthones, and tannins. These compounds play an important role in protecting plants against oxidative stress and pest attacks but also have beneficial effects on human health. The content in total phenols is as high as 17.4% in young leaves but only 13.9% in mature leaves [44]. The main phenolic derivatives in coffee leaves are the esters of caffeic acid with quinic acid (termed chlorogenic acids or CGAs). The most abundant of these esters is 5-caffeoylquinic acid, broadly known as chlorogenic acid. This compound is recognized for its potent antioxidant and anti-inflammatory properties, which are of high relevance for a variety of health issues as well as for skin health and care [41,42]. Research indicates that the levels of chlorogenic acid in coffee leaves are significantly higher than those found in coffee beans, making the leaves a particularly rich source of these beneficial compounds [41,43]. Monteiro et al. conducted a comprehensive analysis of the content of CGAs and other metabolites present in young and mature leaves of four distinct species: *Coffea arabica*, *Coffea canephora*, *Coffea eugenioides*, and *Coffea racemosa*. Their findings showed that, among young leaves, *Coffea arabica* exhibited the highest concentration of CGAs, quantified as 73.5 mg/g dry matter in young leaves, from which 56.8 mg/g were 5-caffeoylquinic acid [45]. In contrast, in mature leaves of *Coffea arabica*, total CGAs were only 19.1 mg/g, of which 5-caffeoylquinic acid was 14.68 mg/g. In highlighting the variability of CGA levels in different developmental stages and species, this study emphasizes the importance of agricultural practices in the obtainment of high-quality plant material determined for later use in applications targeting human health. In addition to caffeic acid, other phenolic acids, like ferulic acid, p-coumaric acid, protocatechuic acid, sinapic acid, and their derivatives, are also present in leaves [43,46].

*Coffea arabica* leaves have been shown to contain several important flavonoids including quercetin, rutin, and kaempferol, among others [22,39,43]. The total flavonoid content of *Coffea arabica* leaves has been quantified, showing that these compounds may constitute a substantial part of the phytochemical profile of the leaves. Specifically, the flavonoid content can vary, but studies have reported values around 0.5% to 1.5% of the dry weight of the leaves [39]. Procyanidins (flavonoid oligomers) were detected by several research groups as well, with procyanidins B and C, procyanidin trimers, and tetramers being identified via HPLC-MS [22,42].

Mangiferin, a phenolic xanthonoid, is a representative compound for *Coffea arabica* leaves, reaching up to 0.2% of the dry weight, depending on the growth region and the specific environmental conditions [22,47]. The concentration of mangiferin in *Coffea arabica* leaves varies. For example, one study reported that in *Coffea arabica* leaves from Brazil and Costa Rica, the total mangiferin content was between 0.6 and 5 mg/g [48]. Research has shown that mangiferin levels tend to decrease with age. In particular, leaves at stages 2 and 3 (leaves at the second and first node below the apex) contain about three-times less mangiferin than the youngest leaves [24].

Diterpenes, including cafestol and kahweol, which are generally found in the lipid fraction of coffee seeds, are also detected in coffee leaves. The concentration of diterpenes in coffee leaves varies significantly depending on the species and growing conditions. Studies have shown that, for example, the concentration of cafestol in *Coffea arabica* leaves is about 0.5 mg/g, while in *Coffea canephora* leaves, it is much lower, being about 0.05 mg/g [49]. Kahweol, another important diterpene, was detected in negligible amounts in *Coffea canephora* leaves but was found in *Coffea arabica* leaves [49]. These data suggest that *Coffea arabica* leaves have a higher content of diterpenes compared to other species, making them a valuable source for further studies on the benefits of these compounds.

In a study conducted by Martins and co-workers on primary metabolites present in *Coffea arabica* leaves, a total of 63 distinct metabolites were found in specimens cultivated in southeast Brazil. Among the carbohydrates identified, the study highlighted the presence of various monosaccharides, including glucose, fructose, galactose, and rhamnose, as well as oligosaccharides such as sucrose and maltose. In addition, polyols such as treitol, sorbitol, and galactinol were detected, along with starch, which plays a crucial role in energy storage within the plant. The amino acid profile was also notable, with the detection of 20 common amino acids, along with β-alanine, homoserine, 4-hydroxyproline, cystine, and gamma-aminobutyric acid (GABA). In addition, polyamines such as ornithine, putrescine, and spermidine were identified, indicating a complex metabolic landscape in *Coffea arabica* leaves [50].

The chemical composition of mature *Coffea arabica* leaves from different regions of Ethiopia shows a remarkable range of macronutrients and minerals. In particular, the protein content ranges from 14.4% to 19.0%, fat content ranges from 4.5% to 12.5%, fiber content is reported to be 17.1% to 20.0% and carbohydrates constitute 51.0% to 63.9% of the leaf composition. This variability is attributed to the diversity of agroecological conditions in different growing regions of Ethiopia, which is recognized as the center of origin for *Coffea arabica* [46,51,52]. In addition, mineral contents, especially magnesium and iron, also vary significantly depending on the specific growing region, emphasizing the influence of local soil composition and environmental factors on the nutritional profile of coffee leaves [53,54]. In particular, the mineral content of Ethiopian coffee leaves, which ranges from 8.8% to 12.4%, is significantly higher than that found in coffee seeds (3.9% to 4.4%) [46]. This suggests that coffee leaves may serve as a more concentrated source of essential minerals compared to other commonly consumed plant materials.

In general, *Coffea arabica* leaves are a promising source of various phytochemicals that possess significant antioxidant, anti-inflammatory, and antimicrobial properties. The rich profile of bioactive compounds, including chlorogenic acids, flavonoids, and alkaloids, positions coffee leaf extracts as valuable ingredients in dermato-cosmetic applications. The synergistic effects of these phytochemicals confer valuable properties to leaf extracts. For example, the combination of flavonoids and phenolic acids may provide improved protection against oxidative stress while promoting skin repair and regeneration. In addition, the anti-inflammatory and antimicrobial properties of these compounds may enhance the efficacy of skin care products to treat various skin conditions, including acne, eczema, and signs of aging [55]. Table 1 presents the chemical structures of key secondary metabolites identified in *Coffea arabica*, presenting their structural diversity. As research continues to explore the effects of these compounds, the potential of *Coffea arabica* to become an important active ingredient in the cosmetics industry becomes increasingly evident. An important aspect of the utilization of coffee leaves is represented by the optimization of extraction methods, as they can significantly influence the yield of bioactive compounds and the composition of extracts. For example, studies have shown that different solvents and extraction techniques can lead to variations in the concentration of chlorogenic acids and other phenolic compounds [14,56].

### 2.2. Phytochemicals in Coffea arabica Seeds

Green coffee beans predominantly comprise various macronutrients and bioactive compounds, with carbohydrates constituting about 59–61% of their total composition. Lipids account for 11–17%, while proteins account for 10–16%. In addition, phenolic compounds account for 6–10%, and minerals contribute about 4%. Other constituents are fatty acids (2%), caffeine (1–2%), trigonelline (1%), and free amino acids, which are present in amounts less than 1%. The roasting process induces notable changes in the composition of green coffee beans. In particular, there is a significant reduction in carbohydrate levels, which fall to 38–42%. Similarly, concentrations of proteins and phenolic compounds decrease, with proteins dropping to 8–14% and phenols to 3–4%. In addition, a decrease in free amino acid levels is also observed. In contrast, changes in lipids, minerals, fatty acids, caffeine, and trigonelline concentrations are relatively insignificant, suggesting that these components are more stable during roasting [57]. The coffee roasting process is a complex thermal treatment that induces a series of chemical transformations, prominently characterized by the Maillard reaction. This reaction is fundamental in the development of flavor and color in roasted coffee, leading to the formation of melanoidins, which are high-molecular-weight compounds that contribute significantly to the sensory attributes of the final product [58,59]. More importantly, melanoidins have antioxidant, antimicrobial, and anti-inflammatory properties that are particularly relevant in a dermato-cosmetic context [37]. These compounds account for about 29% of the total weight of roasted coffee [60]. In addition to the contributions of melanoidins, the lipid fraction of coffee seeds plays an important role as a dermato-cosmetic ingredient. The lipid profile predominantly comprises triacylglycerols, constituting about 75.2% of the total lipid content [61]. This is complemented by esters of diterpene alcohols, which account for about 18.5% of the lipid fraction. Other lipid components include a minor proportion of steroid esters (3.2%), diterpene alcohols (~0.4%), sterols (2.2%), tocopherols (~0.05%), phosphatides (~0.4%), and tryptamine derivatives (~0.8%). Furthermore, the oil extracted by pressing from roasted *Coffea arabica* seeds also contains caffeine, chlorogenic acids, and more than 30 volatiles, with furfurythiol and pyrazines being the main substances. This complex composition not only imprints valuable protective properties against free radicals and UV radiation (with a sun protection of 9.7) but also highly appreciated aroma characteristics [62].

*Coffea arabica* seeds are a rich source of various phytochemicals that have attracted attention for their potential applications in dermato-cosmetic formulations. The main classes of phytochemicals found in *Coffea arabica* seeds include alkaloids, phenolic compounds, lipids, and proteins. Each of these classes contains specific compounds that contribute to the bioactivity of the seeds, particularly in skin health and cosmetic applications.

Alkaloids are one of the most important classes of phytochemicals in *Coffea arabica* seeds, caffeine being the best-known example. Caffeine is recognized for its stimulatory effects and is widely used in cosmetic formulations due to its ability to enhance microcirculation, particularly in eye creams to reduce dark circles [63]. Caffeine concentration shows significant variations between green and roasted seeds of *Coffea arabica* and *Coffea canephora*. Such findings emphasize the importance of considering both genetic and environmental factors when assessing caffeine concentrations in these species. Studies indicate that the reduction in caffeine content can be around 10 to 15 percent after roasting for both *Coffea arabica* and *Coffea canephora*. Theobromine levels tend to decrease more significantly than caffeine levels. The reduction in theobromine content can be around 20 to 30 percent after roasting. Theophylline is usually present in much lower concentrations compared to caffeine and theobromine. Its content may decrease by about 15 to 25 percent during roasting, although specific data may vary. Trigonelline is known to degrade during frying, resulting in a more substantial reduction. The trigonelline content can decrease by about 30 to 50 percent after roasting, particularly in *Coffea arabica* beans. A reduction in the concentration of these alkaloids during the roasting process may affect the flavor and potential health benefits of coffee [24].

Phenolic compounds, particularly chlorogenic acids, are another significant class of phytochemicals in *Coffea arabica* seeds. Chlorogenic acids, which include compounds such as caffeoylquinic acid, are known for their potent antioxidant and anti-inflammatory properties [63,64] The chlorogenic acid (CGA) content of green and roasted *Coffea arabica* beans undergoes significant changes during the roasting process. Studies have shown that roasting green coffee beans can lead to a reduction in CGA content of approximately 30 to 50%.

Diterpenes identified so far in coffee exist predominantly in the form of fatty acid esters, the most notable compounds being cafestol, kahweol, and 16-methoxyfestol (16-WTO). The concentrations of these specific diterpenes show significant variability between different coffee species. In particular, the kahweol content in *Coffea arabica* is significantly higher compared to that found in *Coffea canephora*. Furthermore, it is important to emphasize that 16-WTO is exclusively present in *Coffea arabica*, highlighting the distinct phytochemical profiles of these two species [24,25,26,27,28,29,30,31,32,33,34,35,36,37,38,39,40,41,42,43,44,45,46,47,48,49,50,51,52,53,54,55,56,57,58,59,60,61,62,63,64,65,66,67]. The content of diterpenes, particularly cafestol and kahweol, in green and roasted *Coffea arabica* seeds similarly changes the roasting process. Cafestol is known to be relatively stable during roasting, but some studies indicate that its content may decrease. The reduction in cafestol content after roasting is usually low, around 10 to 20%. The reduction in kahweol content can be more pronounced, with studies indicating a decrease of around 20 to 30% after roasting. In general, although both cafestol and kahweol are present in higher concentrations in green beans, their levels decrease during roasting, which may influence the health benefits associated with these compounds. Specific reductions may vary depending on factors such as temperature and roasting time [24].

Lipids, especially fatty acids, are also present in *Coffea arabica* seeds and contribute to their dermato-cosmetic applications. The lipid profile of *Coffea arabica* seeds includes essential fatty acids and sphingolipids, known for their moisturizing and emollient properties [68].

Proteins, including storage proteins such as globulins, are another important class of phytochemicals in *Coffea arabica* seeds. These proteins can provide structural support and contribute to the seeds’ overall nutritional profile. A protein fraction from defective green seeds was investigated and showed anti-tyrosinase, antimicrobial, and antioxidant activities [69].

The roasting process greatly influences the sensory properties of seeds, enhancing their aroma, which can add value to cosmetic formulations, aiming to create a pleasant sensory experience [62]. Not only the lipid fraction of seeds but also the hydrolates obtained from roasted seeds contain a series of volatiles that imprint a pleasant aroma. Grassino and co-workers showed recently that coffee hydrolates from roasted seeds have a high content of phenols (up to 17%), pyrazines (up to 10%), and aldehydes (up to 7%) [70]. Formulation techniques like microencapsulation provide cosmetics with a good stability against degradation and longer preserved aroma [71]. In addition to the impact of the roasting process, the stage of ripeness of the coffee beans plays an important role in determining the volatile composition.

The differences in chemical composition between green and roasted beans further emphasize the versatility of *Coffea arabica* as an active ingredient in the cosmetics industry, providing opportunities for innovative product development [71].

### 2.3. Comparative Analysis of the Chemical Composition Between the Leaves and Seeds (Green and Roasted) of Coffea arabica

Comparative analysis of the chemical composition between *Coffea arabica* leaves and seeds reveals significant differences in phytochemical profiles, which can be attributed to their distinct biological functions and roles within the plant. Both leaves and seeds are rich in phytochemicals, including alkaloids, phenolic compounds, lipids, and proteins. However, concentrations and specific compounds vary in these plant organs. Table 2 presents a comparative analysis of the concentrations of the primary representative secondary metabolites found in the leaves, green seeds, and roasted seeds of *Coffea arabica*, highlighting the variations in metabolite profiles across these different plant parts.

Different concentrations of secondary metabolites are observed in *Coffea arabica* leaves and seeds. Thus, caffeine is present both in seeds and leaves of *Coffea arabica*, with the highest concentrations in green seeds (7.6–29.0 mg/g) and lower in leaves (2.5–6.0 mg/g) [24]. After roasting, the caffeine content remains relatively stable, with a reduction of about 10% to 15%, resulting in about 1.0% to 1.35% of the dry matter for roasted *Coffea arabica* beans [72]. The trigonelline content in leaves is generally lower than in seeds. The abundance is highest in green seeds (8.8–27.6 mg/g), but after roasting, it is drastically reduced. Phenolic compounds, especially chlorogenic acids, are another important class found in both leaves and seeds. In *Coffea arabica* seeds, chlorogenic acids usually account for 5–8% of the dry weight [73]. In leaves, they reach a concentration of 19.2–39.6 mg/g [24]. In roasted seeds, the levels decrease substantially due to the degradation of simpler compounds. Mangiferin is only detected in leaves being completely absent in green or roasted seeds. Regarding the presence of diterpenes, cafestol is more abundant in green seeds (2.7–11.0 mg/g) compared to roasted seeds and is present in limited amounts in leaves [24], while kahweol is detected only in seeds and is absent from leaves.

The concentration of the main primary metabolites emphasizes the different biological functions between leaves and seeds. Table 3 presents a comparative analysis of the concentrations of the primary representative metabolites in the leaves, green seeds, and roasted seeds of *Coffea arabica*, illustrating the differences in metabolite levels among these distinct plant components. Carbohydrate levels are similar in green leaves and seeds but decrease after roasting (38–42%) due to Maillard reactions [57]. Lipids are another class of phytochemicals present in both leaves and seeds, but their composition and concentration differ significantly. In *Coffea arabica* seeds, lipids typically constitute between 11% and 17% of the dry weight [57]. These lipids include essential fatty acids, which are known for their moisturizing properties and ability to maintain the skin’s barrier function, making them beneficial in cosmetic formulations for dry or sensitive skin. In contrast, the lipid content of leaves is generally lower, around 5% to 10% of dry weight, and consists mainly of phospholipids and glycolipids, which play roles in cell structure and function rather than in cosmetic applications [74]. In *Coffea arabica* seeds, proteins typically account for between 10% and 16% of dry weight [57]. These proteins, including storage proteins such as globulins, contribute to the nutritional profile of the seeds and can enhance skin elasticity and firmness when used in cosmetic formulations. In contrast, the protein content of leaves is generally lower, often between 5% and 8% of dry weight, and includes various enzymes and structural proteins that support the metabolic functions of leaves [74].

In general, comparative analyses of the chemical composition of *Coffea arabica* leaves and seeds reveal distinct phytochemical profiles, with significant differences in concentrations and types of compounds. The seeds are rich in alkaloids, especially caffeine, and contain substantial amounts of polysaccharides, lipids, and proteins, making them valuable for energy storage and cosmetic applications. The roasting process further changes the chemical profile of the seeds, increasing some flavor compounds and reducing others. In contrast, the leaves are richer in phenolic compounds and have a different lipid and protein profile, highlighting their role in plant defense and metabolic processes. Understanding these differences is essential for the effective use of *Coffea arabica* in dermato-cosmetic applications, allowing the development of products that capitalize on the unique properties of both leaves and seeds.

**Figure 2:**
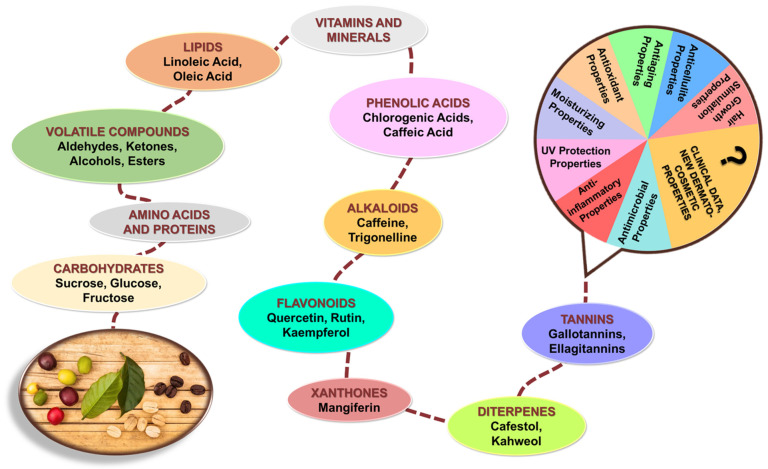
Comprehensive overview of the primary and secondary metabolites in *Coffea arabica*, their representative compounds, and their potential roles in dermato-cosmetic applications. The chart also highlights the bioactive properties attributed to these metabolites, emphasizing their relevance in skin care. Source of image depicting coffee leaves and seeds: https://www.alamy.com/cofffea-fruits-leaves-roasted-coffee-beans-image362695248.html, accessed on 26 November 2024.

Figure 2 illustrates the diverse phytochemical profile of *Coffea arabica*, focusing on both primary and secondary metabolites and their properties in dermato-cosmetic applications. The diagram further links these compounds to their dermato-cosmetic benefits, such as antioxidant, UV protection, anti-inflammatory, anti-aging, moisturizing, and antimicrobial effects. Additionally, the chart underscores the need for further clinical research to explore and validate new dermato-cosmetic applications.

## 3. Dermato-Cosmetical Effects of *Coffea arabica*

### 3.1. Anticellulite Properties

Cellulite, commonly referred to as gynoid lipodystrophy, is a prevalent condition affecting 80–90% of postpubertal women, characterized by a dimpled appearance and an uneven skin texture resembling “orange peel” [75,76]. Its formation is attributed to various factors, including structural, hormonal, and vascular changes in subcutaneous adipose tissue. It is primarily caused by the accumulation of fat beneath the skin, changes in connective tissue, and fluid retention [76,77].

The anticellulite properties of *Coffea arabica* have gained attention in the field of dermato-cosmetics due to the presence of various bioactive compounds in its leaves and seeds. The application of *Coffea arabica* extracts, particularly those rich in caffeine, chlorogenic acids, and flavonoids, has been shown to effectively address these issues.

Caffeine found in *Coffea arabica* has emerged as a significant ingredient in anticellulite formulations due to its lipolytic properties. Caffeine has a lipolytic effect via several mechanisms. Its main mechanism of action involves the antagonization of adenosine receptors, which increases cyclic AMP (cAMP) levels in fat cells. It stimulates lipolysis, the breakdown of fat cells in adipocytes, and inhibits phosphodiesterase enzymes, which are responsible for fat accumulation [78]. It also improves microcirculation in the skin. Clinical studies have indicated that the topical application of caffeine can lead to a reduction in the appearance of cellulite by enhancing blood flow, promoting the drainage of excess fluid from the affected areas and promoting collagen synthesis [79,80]. Concentrations of caffeine in topical formulations typically range from 1% to 5%, with higher concentrations often yielding more pronounced effects. For example, a study demonstrated that a cream containing 3% caffeine significantly improved skin texture and reduced the appearance of cellulite in clinical trials [79]. Coffee extracts can vary in caffeine percentage, and advanced processing techniques such as solvent extraction or supercritical extraction are required to achieve high caffeine concentrations [81]. Furthermore, formulations combining caffeine with other active ingredients, such as retinol and herbal extracts, have shown improved efficacy in cellulite treatment [80,82]. To increase the effectiveness of caffeine in the treatment of cellulite, herbal extracts with complementary properties can be used: green tea extract, rich in catechins, stimulates metabolism and fat burning; *Ginkgo biloba* extract improves blood circulation, reducing the appearance of cellulite; algae extract detoxifies the skin and improves its elasticity by providing minerals and antioxidants. These extracts contribute synergistically by stimulating circulation, reducing inflammation, and optimizing cellular metabolism, amplifying the effects of caffeine in the fight against cellulite [82].

Chlorogenic acids (CGAs), another key group of phytochemicals in *Coffea arabica*, exhibit significant potential as anticellulite agents due to their multifaceted mechanisms of action. These polyphenolic compounds are known to enhance antioxidant defenses, thereby mitigating oxidative stress, which is a contributing factor to cellulite formation [83]. It has been shown to inhibit the activity of enzymes that contribute to the breakdown of collagen and elastin in the skin, which are crucial for maintaining skin firmness and elasticity [84]. CGAs also influence lipid metabolism by inhibiting enzymes involved in cholesterol synthesis, such as hydroxymethylglutaryl-CoA reductase, which may help in reducing fat accumulation in adipose tissues [85]. Moreover, CGAs have been shown to increase cell membrane permeability, facilitating the release of stored lipids and promoting fat mobilization [86]. Their anti-inflammatory properties further support skin health by reducing inflammation associated with cellulite [83]. Formulations incorporating CGAs can enhance skin elasticity and hydration, contributing to an overall improvement in skin texture. Thus, the incorporation of chlorogenic acids into topical formulations presents a promising strategy for cellulite management. The concentration of chlorogenic acids in *Coffea arabica* extracts can vary, but formulations containing 1% to 3% of these acids have been reported to effectively improve skin tone and texture, thereby reducing the visibility of cellulite [84].

Flavonoids derived from *Coffea arabica* exhibit significant potential as anticellulite agents due to their antioxidant and anti-inflammatory properties. These compounds have been shown to reduce oxidative stress and inflammation, which are major factors in cellulite formation [84,87]. The mechanism of action involves the inhibition of matrix metalloproteinase-1 (MMP-1), an enzyme that contributes to collagen degradation, thereby promoting skin elasticity and firmness [84]. They are well known for their ability to enhance vascular health and reduce inflammation, which can contribute to improved skin appearance. Flavonoid concentrations in *Coffea arabica* extracts typically range from 0.5% to 2%, and their inclusion in formulations has been associated with enhanced skin hydration and elasticity, further aiding in reducing cellulite [88]. In topical formulations, such as creams and gels, *Coffea arabica* extracts can enhance skin penetration and stability, allowing for the effective delivery of these bioactive compounds [89]. The antioxidant activity of flavonoids helps to scavenge free radicals, further supporting skin health and potentially improving the appearance of cellulite [73]. Additionally, the anti-inflammatory effects of these compounds can alleviate skin irritation and promote a smoother texture [41,87].

The extract type also influences the efficacy of *Coffea arabica* in anticellulite applications. For instance, aqueous extracts derived from the leaves tend to retain higher levels of chlorogenic acids and flavonoids compared to extracts from roasted seeds, which may lose some of these compounds during the roasting process. Therefore, using green coffee extracts or minimally processed extracts can enhance the overall effectiveness of anticellulite formulations [88].

In addition to the individual compounds, the synergistic effects of these phytochemicals in *Coffea arabica* contribute to their anticellulite properties. For example, formulations that combine caffeine with chlorogenic acids and flavonoids have been shown to provide enhanced results compared to those containing a single active ingredient. This synergy can lead to improved lipolysis, reduced inflammation, and enhanced skin elasticity, all of which are critical for combating cellulite [79].

Moreover, the stability of these active compounds in formulations plays a significant role in maintaining their efficacy over time. Research indicates that formulations containing *Coffea arabica* extracts should be stored in opaque containers to protect them from light, which can degrade sensitive compounds like chlorogenic acids. Additionally, maintaining a lightly acidic pH in formulations can help preserve the integrity of these bioactive compounds, ensuring their effectiveness upon application [88].

### 3.2. Anti-Aging Properties

The aging process of the skin is a multifaceted phenomenon characterized by intrinsic and extrinsic factors. Intrinsic aging, or chronological aging, leads to structural changes such as decreased collagen production, loss of elasticity, and increased dryness, manifesting as wrinkles and sagging skin [73,89]. Extrinsic aging, primarily driven by environmental factors like UV exposure and pollution, exacerbates these changes, resulting in photoaging, which is marked by pigmentation irregularities and further collagen degradation [90,91].

*Coffea arabica*, known for its antioxidant, anti-inflammatory, and skin-rejuvenating properties, has been explored as an anti-aging ingredient in various formulations, making *Coffea arabica* a promising ingredient in formulations aimed at combating the visible signs of aging. Its bioactive compounds can mitigate oxidative stress, a significant contributor to skin aging [92,93]. Studies suggest that formulations containing *Coffea arabica* can enhance skin hydration and elasticity, potentially counteracting the visible signs of aging, through mechanisms that include the following: stimulating microcirculation, thus increasing blood flow; delivering essential nutrients and oxygen to the skin, through antioxidant properties, which protect skin cells from oxidative stress; maintaining elasticity as well as reducing inflammation; reducing edema, contributing to an even texture [92,94]. The incorporation of this natural extract into topical applications may provide a synergistic effect, promoting skin health by protecting against free radical damage and supporting collagen synthesis [92,95].

Chlorogenic acids (CGAs), particularly 5-caffeoylquinic acid (5-CQA) derived from *Coffea arabica*, exhibit significant anti-aging properties through their antioxidant and anti-inflammatory mechanisms. These polyphenolic compounds are known to mitigate oxidative stress by scavenging free radicals, thereby reducing cellular damage associated with skin aging [41,96,97]. Additionally, CGAs have been shown to inhibit pro-inflammatory cytokines such as TNF-α, which are implicated in skin aging and inflammatory responses [87]. Studies have shown that formulations containing 1% to 3% chlorogenic acids can effectively inhibit lipid peroxidation and protect skin cells from UV-induced damage, as they possess photostability under UV exposure, making them suitable for incorporation into sunscreens [41,98]. The ability of chlorogenic acids to modulate the expression of matrix metalloproteinases (MMPs), which are involved in collagen degradation, further supports their role in maintaining skin elasticity and firmness, contributing to a more youthful appearance [97,99]. Overall, the incorporation of CGAs from *Coffea arabica* into various formulations can provide multifaceted benefits, promoting skin health and longevity.

Caffeine, derived from *Coffea arabica*, exhibits multiple mechanisms of action that contribute to its efficacy as an anti-aging ingredient in various formulations. Primarily, caffeine possesses potent antioxidant properties, which protect skin cells from oxidative stress and ultraviolet (UV) radiation, thereby mitigating photoaging effects [82,100,101]. Additionally, caffeine promotes microcirculation and enhances the delivery of nutrients to skin cells, which can improve skin tone and texture, improving overall skin appearance [82,101]. Topical formulations containing caffeine concentrations ranging from 1% to 5% have been shown to reduce the appearance of fine lines and wrinkles by promoting collagen synthesis and inhibiting the breakdown of existing collagen [102]. Additionally, caffeine’s ability to reduce inflammation contributes to its effectiveness in anti-aging products. Topically applied caffeine has been shown to absorb UV radiation, acting as a sunscreen and providing an additional layer of protection against skin cancer [103,104]. Furthermore, caffeine’s ability to activate autophagy contributes to the elimination of reactive oxygen species (ROS), promoting skin rejuvenation and reducing senescence [100]. The formulation of caffeine in nanoemulsions or solid lipid nanoparticles enhances its skin penetration, maximizing its therapeutic effects [105,106]. Collectively, these mechanisms underscore caffeine’s role as a valuable anti-aging ingredient in cosmetic formulations.

Flavonoids derived from *Coffea arabica* exhibit significant anti-aging properties through various mechanisms, primarily due to their potent antioxidant and anti-inflammatory activities. These compounds can protect skin cells from oxidative damage and reduce inflammation, which are critical factors in the aging process [84,107]. The antioxidant capacity of these flavonoids has been demonstrated to inhibit matrix metalloproteinases (MMPs), which are enzymes that degrade collagen and elastin in the skin, further promoting skin elasticity and reducing wrinkles [84,102]. Formulations containing 0.5% to 2% flavonoids have been shown to enhance skin hydration and elasticity, further supporting their use in anti-aging products [108]. Formulations incorporating these compounds, such as creams and gels, leverage their bioactive properties to improve skin hydration and reduce inflammation, thereby contributing to a more youthful appearance [109]. The integration of *Coffea arabica* in cosmetic products not only provides immediate benefits but also supports long-term skin health through its multifaceted action against aging [105].

The presence of various phenolic compounds in *Coffea arabica* contributes to its overall antioxidant capacity. These compounds, including chlorogenic acid and caffeic acid, function as free radical scavengers, effectively neutralizing the activity of enzymes involved in the degradation of collagen and elastin, thereby promoting skin firmness and reducing the appearance of sagging skin [107,110]. Concentrations of phenolic compounds in formulations typically range from 1% to 3%, providing effective protection against oxidative stress [111].

*Coffea arabica* also contains proteins and peptides, such as 12S globulin, that can enhance skin hydration and support the skin’s structural integrity. These compounds can stimulate the production of collagen and elastin, which are essential for maintaining skin elasticity and firmness. The incorporation of protein extracts in concentrations of 1% to 5% in topical formulations has been shown to improve skin texture and reduce the appearance of wrinkles [112,113].

The efficacy of *Coffea arabica* in anti-aging applications can also be influenced by the type of extract used. Aqueous extracts from the leaves tend to retain higher concentrations of chlorogenic acids and flavonoids compared to extracts from roasted seeds, which may lose some beneficial compounds during the roasting process. Therefore, using green coffee extracts or minimally processed extracts can enhance the overall effectiveness of anti-aging formulations [17].

### 3.3. Antioxidant and UV-Protective Properties

Oxidative stress in the skin is mainly caused by an imbalance between reactive oxygen species (ROS) and the body’s antioxidant defenses, leading to cell damage and accelerated aging. Environmental factors such as UV radiation and pollution amplify this process, generating free radicals that damage collagen, DNA, and other cellular structures [114]. Antioxidants are important in neutralizing these free radicals, preventing oxidative damage [115,116]. *Coffea arabica*, especially its leaves, is rich in chlorogenic acids, flavonoids, and mangiferin, which have antioxidant and anti-inflammatory properties [16,87]. These compounds help to scavenge free radicals and reduce inflammation, promoting skin health [14,117]. Cosmetic formulations that include *Coffea arabica* may enhance skin protection against oxidative stress and aging [118]. Chlorogenic acids (CGAs), particularly 5-caffeoylquinic acid (5-CQA), are among the most abundant antioxidants in *coffee arabica*. These compounds neutralize reactive oxygen species, prevent lipid peroxidation, and chelate metal ions, inhibiting the Fenton reaction [41,63,119]. Chlorogenic acids reduce oxidative stress and inflammation, protecting collagen and preventing oxidative damage [41,84]. Studies show that chlorogenic acid concentrations up to 1% significantly reduce lipid peroxidation [41,63]. CGA protects against UV-induced damage and promotes wound healing by increasing levels of superoxide dismutase and catalase [46,120]. Formulations including CGA, such as microemulsions, provide improved penetration and protective effects on the skin [121]. Caffeine derived from *Coffea arabica* has remarkable antioxidant and photoprotective properties. This metabolite inhibits lipid peroxidation, protects cell membranes [122], and increases the expression of antioxidant enzymes such as superoxide dismutase (SOD) and catalase [123]. In concentrations of 1–5%, it provides effective protection against oxidative damage and improves skin resistance [124]. Caffeine also acts as a physical UV filter, absorbing UV radiation and supporting skin protection [103,104]. Caffeine inhibits UV-induced apoptosis and promotes the elimination of precancerous cells by activating autophagy [100,125]. Flavonoids in *Coffea arabica* leaves can scavenge free radicals and increase skin elasticity [63,99,117]. Flavonoid concentrations between 0.5 and 2% increase the antioxidant capacity of cosmetic formulations, protecting against skin aging [63]. Flavonoids absorb UV radiation and provide additional protection against skin damage [112,126]. Regarding the photoprotection provided by *Coffea arabica*, polyphenols, including chlorogenic acids and flavonoids, have been shown to inhibit ROS generated by UV radiation [84,127]. *Coffea arabica* extracts reduce the levels of inflammatory markers (TNF-α) and support cell repair processes [87,128]. Studies indicate an increased potential for skin UV protection when extracts are incorporated into natural sunscreens [127,129]. *Coffea arabica* extracts are notable for their good stability in neutral media, and the use of opaque containers can significantly contribute to preventing their degradation [41]. Minimally processed extracts, such as ultrasound-assisted extracts, optimize their efficiency and antioxidant activity. Both coffee seeds and leaf extracts have antioxidant potential, but their effectiveness may vary depending on the chemical composition and extraction method used [41,130].

### 3.4. Anti-Inflammatory Properties

The inflammatory process of the skin is a complex response to various stimuli, including infections and oxidative stress, which can lead to chronic skin conditions if dysregulated. Chronic inflammation is a significant contributor to various skin conditions, including acne, eczema, and psoriasis. Key inflammatory mediators such as TNF-α and IL-6 are involved in this process, contributing to skin damage and aging [131,132]. In this context, natural ingredients with anti-inflammatory properties, such as those derived from *Coffea arabica*, have gained attention for their potential therapeutic benefits in skin care formulations.

*Coffea arabica* contains bioactive compounds that exhibit significant anti-inflammatory effects, potentially reducing the expression of inflammatory cytokines and mediators. Its antioxidant properties further enhance skin protection by neutralizing reactive oxygen species (ROS), which are implicated in skin inflammation and aging [109]. Formulations incorporating *Coffea arabica* can, thus, provide dual benefits, alleviating inflammation while promoting skin health through antioxidant activity. For instance, a study demonstrated that an aqueous extract of *Coffea arabica* leaves exhibited high anti-inflammatory potential by significantly reducing the levels of inflammatory markers in skin cells exposed to inflammatory stimuli [133]. This makes it a promising candidate for developing effective skin care products aimed at managing inflammatory skin conditions.

Chlorogenic acids, particularly from *Coffea arabica*, exhibit significant anti-inflammatory properties that can be beneficial in various skin formulations. These compounds have been demonstrated to possess significant anti-inflammatory effects, primarily through the inhibition of pro-inflammatory cytokines such as tumor necrosis factor-alpha (TNF-α) and interleukin-6 (IL-6) [63]. They can modulate inflammatory pathways by suppressing key mediators, including cyclooxygenase-2 (COX-2), nuclear factor kappa-light-chain-enhancer of activated B cells (NF-κB), and inducible nitric oxide synthase (iNOS), which play critical roles in skin inflammation and wound healing processes [88,134]. Research indicates that topical formulations containing 1% to 3% chlorogenic acids can effectively reduce inflammation in skin cells exposed to inflammatory stimuli [63]. Recent studies have demonstrated that *Coffea arabica* extracts, rich in chlorogenic acids, can alleviate conditions like atopic dermatitis by regulating inflammasome expression and improving skin barrier functions [135]. Additionally, formulations containing these extracts have been shown to reduce lipid peroxidation and enhance collagen synthesis, contributing to overall skin health [41,84]. Thus, incorporating chlorogenic acids into skin care products can leverage their dual role as potent anti-inflammatory and antioxidant agents, promoting skin healing and protection against environmental stressors [83,88,136].

Caffeine derived from *Coffea arabica* exhibits notable anti-inflammatory properties. Caffeine has demonstrated anti-inflammatory properties through its ability to inhibit phosphodiesterase, leading to increased levels of cyclic adenosine monophosphate (cAMP). Elevated cAMP levels can reduce the production of pro-inflammatory mediators and enhance the skin’s barrier function [137]. Its ability to enhance the levels of anti-inflammatory cytokines, such as interleukin-10 (IL-10), suggests a beneficial role in mitigating inflammation, particularly in response to physical stressors [138,139]. Caffeine’s hydrophilic nature allows for effective penetration through the skin barrier, enhancing local microcirculation and promoting skin health [101,140]. Studies have demonstrated that formulations containing caffeine can also inhibit lipid peroxidation, further supporting its anti-inflammatory and protective effects against skin damage [41,88]. Thus, incorporating caffeine from *Coffea arabica* into skin care products can provide dual benefits of anti-inflammatory and antioxidant effects, promoting overall skin health.

Flavonoids derived from *Coffea arabica* exhibit significant anti-inflammatory properties. These compounds, including quercetin and rutin, are known for their ability to reduce inflammatory markers such as tumor necrosis factor-alpha (TNF-α) and interleukin-6 (IL-6), as well as to inhibit the activity of cyclooxygenase (COX) and lipoxygenase (LOX) enzymes, which are critical in skin inflammation and aging processes [87,117].

### 3.5. Emollient, Skin Hydration, and Moisturization Properties

Emollients play a major role in skin hydration by forming a barrier that reduces transepidermal water loss (TEWL), thereby enhancing skin moisture retention and overall hydration [141,142]. The skin’s hydration status is vital for maintaining its mechanical properties, as well-hydrated skin appears smoother and healthier [143,144]. *Coffea arabica* extracts are the ingredients of numerous patented formulations with moisturizing and emollient effects. These extracts were mainly obtained from coffee seed oil, coffee pulp, and crushed stem cell extract [145].

Research indicates that *Coffea arabica* extracts can contribute to skin hydration by promoting moisture retention and providing a soothing effect, which is essential for maintaining skin integrity [146]. Additionally, the presence of an array of bioactive compounds in *Coffea arabica* may help combat oxidative stress, further supporting skin health and hydration [14]. Therefore, incorporating *Coffea arabica* into skin care formulations can enhance their moisturizing efficacy and overall skin benefits.

The emollient and moisturizing properties of *Coffea arabica* are primarily attributed to its lipid content, which includes essential fatty acids and various phytosterols. The lipids extracted from *Coffea arabica* beans comprise 11–17% of their total chemical composition [57]. These lipids play a crucial role in maintaining skin barrier function by forming a protective layer on the skin’s surface, thereby preventing moisture loss and enhancing skin smoothness [126]. The presence of fatty acids such as oleic acid and linoleic acid in *Coffea arabica* seed oil has been shown to improve skin hydration and elasticity, being identified as an effective emollient and reducing transepidermal water loss [147]. An additional contributing mechanism is the effect of coffee seed oil, in particular, obtained from green seeds, on the components of the extracellular matrix and aquaporin expression. Green coffee oil was able to induce the expression of the mRNA of aquaporin-3 to 6.5-fold in comparison to control cultures [148]. Formulations containing 5% to 10% *Coffea arabica* seed oil can effectively enhance the emollient properties of creams and lotions, providing a soft and supple feel to the skin [149].

One of the most significant classes of compounds in *Coffea arabica* is chlorogenic acids (CGAs). These compounds have been shown to modulate the expression of aquaporins, which are water channel proteins that facilitate water transport across cell membranes [150]. By enhancing aquaporin-3 levels in human fibroblasts, chlorogenic acids can significantly improve skin hydration. Concentrations of 1% to 3% of chlorogenic acids in topical formulations have been reported to effectively increase skin hydration levels [53,117,135]. The presence of CGAs in formulations can improve skin barrier function, leading to increased moisture retention and reduced transepidermal water loss [126,149]. Moreover, CGAs possess anti-inflammatory properties that can soothe irritated skin, making them suitable for sensitive skin formulations [86,117]. Their ability to modulate skin hydration is also supported by studies indicating that CGAs can enhance collagen and elastin production, contributing to skin elasticity and firmness [126,149]. Thus, incorporating chlorogenic acids from *Coffea arabica* into skin care products not only provides hydration but also promotes skin health through their multifaceted bioactive properties.

Caffeine, another key component of *Coffea arabica*, not only acts as a stimulant but has also been shown to enhance microcirculation in the skin, which can improve nutrient delivery and hydration. Topical formulations containing caffeine concentrations of 1% to 5% have demonstrated significant improvements in skin moisture retention and overall hydration levels [109]. Its ability to enhance skin hydration is attributed to its role in reducing transepidermal water loss (TEWL), thereby improving the skin barrier function [151,152]. Caffeine also promotes the proliferation of skin cells, which contributes to a more hydrated and rejuvenated appearance [151]. Moreover, the antioxidant properties of caffeine can protect the skin from oxidative stress, further supporting skin health and hydration [152]. Additionally, caffeine’s anti-inflammatory effects can soothe irritated skin, enhancing overall skin texture and moisture retention [153].

Flavonoids derived from *Coffea arabica* exhibit significant emollient, skin hydration, and moisturizing properties, making them valuable ingredients in various cosmetic formulations. These compounds exhibit anti-inflammatory properties that can help soothe irritated skin and support the skin’s natural moisture barrier, as well as acting as potent antioxidants, which help to mitigate oxidative stress in skin cells, thereby enhancing skin barrier function and moisture retention [84,102]. Formulations containing 0.5% to 2% flavonoids have been shown to enhance skin hydration and reduce transepidermal water loss (TEWL), thereby improving overall skin moisture levels [135]. Flavonoids can regulate proteins, with a major effect in maintaining the epidermal barrier, effectively reducing transepidermal water loss, which is essential for skin hydration. Moreover, the presence of phenolic compounds further contributes to the moisturizing effects by promoting collagen synthesis and inhibiting melanin production, thus improving overall skin texture and appearance [84,102]. The incorporation of *Coffea arabica* extracts in formulations not only enhances hydration but also provides protective benefits against environmental stressors, thereby supporting skin health and longevity [84,112].

Moreover, *Coffea arabica* contains proteins and peptides that can enhance skin hydration by attracting and retaining moisture. The moisturizing effects are further supported by the ability of its proteins to bind water, thereby improving skin hydration levels [154]. These compounds can also stimulate collagen and elastin production, which are essential for maintaining skin elasticity and firmness. Concentrations of 1% to 5% of protein extracts in formulations can significantly improve skin hydration and texture [126,135].

### 3.6. Antimicrobial Properties

The microbial infection process of the skin involves the invasion of pathogens, leading to inflammation and potential tissue damage. This process is often exacerbated by compromised skin barriers, which can result from conditions like atopic dermatitis, where oxidative stress and immune dysfunction play significant roles [135].

*Coffea arabica*, particularly its extracts, has demonstrated promising antimicrobial properties, making it a valuable ingredient in various formulations. Studies have shown that *Coffea arabica* extracts exhibit significant antibacterial and antifungal activities due to their high phenolic content, which correlates with antioxidant and anti-inflammatory effects [63,87,88]. Furthermore, these extracts have been found to regulate inflammatory responses and enhance skin barrier functions, thereby potentially alleviating conditions like atopic dermatitis [87,135]. The presence of compounds such as chlorogenic acid and caffeic acid in *Coffea arabica* contributes to its efficacy in promoting skin health and combating microbial infections [87,155].

The phytocompounds present in *Coffea arabica*, such as chlorogenic acid, caffeine, and flavonoids, have been shown to possess notable antimicrobial activities. For instance, studies have demonstrated that extracts from *Coffea arabica* can inhibit the growth of various bacteria and fungi, including skin pathogens. A recent study highlighted that naringin, a compound found in coffee, exhibited inhibitory effects against *Aspergillus fumigatus*, a significant fungal pathogen associated with skin infections and respiratory diseases [155].

Polyphenolic acids derived from *Coffea arabica* exhibit significant antimicrobial properties that can be harnessed in various skin formulations. Chlorogenic acid, the most abundant phenolic compound in *Coffea arabica*, has been shown to possess antibacterial effects against pathogens commonly associated with skin infections such as *Staphylococcus aureus* and *Escherichia coli*, making it a valuable ingredient in topical applications aimed at skin health and wound healing [88,110,120]. For instance, a study demonstrated that an aqueous extract of *Coffea arabica* leaves exhibited high antimicrobial potential against *Staphylococcus aureus* and *Escherichia coli*, significantly reducing their viability at concentrations as low as 0.5% [63,156]. Additionally, the antioxidant properties of polyphenolic compounds help mitigate oxidative stress, thereby enhancing skin barrier function and reducing inflammation [157]. Studies have shown that chlorogenic acid concentrations as low as 0.5% can inhibit the growth of these bacteria, making it a valuable ingredient in formulations aimed at preventing bacterial infections on the skin [41].

Caffeine has also been shown to possess antimicrobial properties. Research indicates that caffeine can inhibit the growth of certain fungi, including *Candida albicans*, which is relevant for skin conditions such as candidiasis. Concentrations of caffeine ranging from 1% to 5% have been effective in reducing fungal growth in vitro. The mechanism of action is believed to involve the disruption of fungal cell membranes, leading to cell death [158].

The flavonoids present in *Coffea arabica* exhibit strong antimicrobial activity. These compounds have been shown to be effective against a variety of bacteria and fungi, including *Propionibacterium acnes*, the bacteria responsible for acne. Formulations containing flavonoid concentrations of 0.5% to 2% have demonstrated significant reductions in bacterial counts, thereby supporting their use in acne treatment products [155].

Moreover, the antifungal activity of *Coffea arabica* extends to other pathogens such as *Candida albicans* and *Aspergillus niger*. Research indicates that mangiferin, a bioactive compound extracted from coffee leaves, has shown antifungal effects against these pathogens. This is particularly relevant given the increasing prevalence of fungal infections, which pose significant health risks, especially in immunocompromised individuals. The ability of *Coffea arabica* to inhibit these pathogens suggests its potential as a natural alternative to synthetic antifungal agents [56].

In addition to its antifungal properties, *Coffea arabica* has demonstrated antibacterial effects against various bacterial strains. For example, an extract from *Coffea arabica* seeds was tested against *Enterococcus faecalis*, a bacterium commonly associated with skin infections, and showed significant antibacterial activity [159]. This highlights the relevance of *Coffea arabica,* not only in treating fungal infections but also in addressing bacterial skin infections, which are often resistant to conventional antibiotics.

The antimicrobial properties of *Coffea arabica* can be attributed to its rich composition of phenolic compounds, which are known for their ability to disrupt microbial cell membranes and inhibit enzyme activity essential for microbial growth. The phenolic compounds formed during the roasting of coffee, such as ketones and aldehydes, have been reported to possess broad-spectrum antimicrobial activities against both Gram-positive and Gram-negative bacteria [160]. This broad-spectrum activity is important in the context of rising antibiotic resistance, as it opens avenues for the use of *Coffea arabica* extracts in developing new antimicrobial therapies.

The application of *Coffea arabica* extracts in dermatological formulations is supported by their ability to inhibit the growth of skin pathogens such as *Trichophyton mentagrophytes* and *Trichophyton rubrum*, which are responsible for common skin infections like athlete’s foot and ringworm [161]. The antifungal activity of spent coffee ground extracts against these pathogens further emphasizes the potential of utilizing coffee waste in developing sustainable antimicrobial products.

### 3.7. Follicle and Hair Growth Stimulation Properties

Hair loss and follicle regeneration are complex biological processes characterized by the cyclic phases of hair growth (anagen), regression (catagen), and rest (telogen) [162,163]. The regeneration of hair follicles involves various signaling pathways, notably the Wnt signaling pathway, which plays a crucial role in hair follicle development and regeneration [164,165]. Recent studies have highlighted the potential of natural ingredients, such as *Coffea arabica*, in promoting hair growth and follicle stimulation [166]. Extracts from *Coffea arabica* have demonstrated antioxidant properties and the ability to enhance hair growth by improving the health of hair follicles and reducing hair loss [109,135]. Clinical trials have shown that formulations containing *Coffea arabica* can significantly increase hair volume and thickness while decreasing hair loss in patients suffering from various forms of alopecia [167,168]. These beneficial effects are mostly attributed to the presence of phenolic compounds in *Coffea arabica*, which support the regeneration of hair follicles and improve overall scalp health [169,170].

Caffeine is one of the most studied compounds in *Coffea arabica* concerning hair growth and has garnered significant attention in the field of dermatology and trichology due to its potential to stimulate hair growth and counteract hair loss, particularly in conditions such as androgenetic alopecia. The mechanisms by which caffeine exerts these effects are multifaceted, involving biochemical pathways that enhance follicular activity and promote hair shaft elongation. One of the primary mechanisms through which caffeine promotes hair growth is its ability to inhibit phosphodiesterase (PDE) enzymes. This inhibition leads to an increase in intracellular cyclic adenosine monophosphate (cAMP) levels, which is crucial for cellular metabolism and proliferation. Elevated cAMP levels have been shown to stimulate the proliferation of keratinocytes, the primary cell type found in hair follicles, thereby enhancing hair growth [101,171,172]. Additionally, caffeine has been demonstrated to counteract the inhibitory effects of dihydrotestosterone (DHT), a potent androgen that contributes to hair follicle miniaturization and subsequent hair loss. By inhibiting PDE, caffeine effectively mitigates the negative impact of DHT on hair follicles, promoting a healthier growth environment [171,173]. Research has indicated that caffeine not only stimulates hair follicle proliferation but also prolongs the anagen phase of the hair cycle, which is the active growth phase. This effect is particularly significant as a longer anagen phase correlates with increased hair density and length [174,175]. Studies have shown that caffeine enhances hair shaft elongation and promotes the proliferation of hair matrix keratinocytes, which are essential for hair growth. Moreover, caffeine has been found to increase the expression of insulin-like growth factor 1 (IGF-1), a known promoter of hair follicle growth, while simultaneously downregulating transforming growth factor-beta (TGF-β), which is associated with hair growth inhibition [175,176]. The bioavailability of caffeine in the scalp is another critical factor influencing its effectiveness in promoting hair growth. Caffeine’s hydrophilic nature allows it to penetrate the lipid skin barrier effectively, reaching the hair follicles where it can exert its effects [140]. Various formulations, including topical applications and nanostructured lipid carriers, have been developed to enhance the delivery of caffeine to hair follicles, thereby maximizing its therapeutic potential [177]. For instance, studies have shown that optimized formulations can significantly improve the penetration of caffeine into the hair follicles, leading to enhanced hair retention and growth [178]. Furthermore, caffeine’s role in improving local microcirculation in the scalp is an essential aspect of its hair growth-promoting properties. By increasing blood flow to the hair follicles, caffeine ensures that these follicles receive adequate nutrients and oxygen, which are vital for healthy hair growth [101,140]. This enhanced microcirculation not only supports the metabolic needs of the hair follicles but also aids in the removal of metabolic waste products, creating a more favorable environment for hair growth. Studies have demonstrated that topical formulations containing caffeine concentrations of 1% to 5% can significantly enhance hair growth in both in vitro and in vivo models [179,180]. The mechanism involves the promotion of keratinocyte proliferation and the inhibition of apoptosis in dermal papilla cells (DPCs), which are critical for hair growth [167]. In clinical settings, caffeine-based treatments have shown promising results in comparison to traditional therapies such as minoxidil. A randomized controlled trial demonstrated that a caffeine-based topical solution was non-inferior to minoxidil in treating male androgenetic alopecia, highlighting caffeine’s efficacy as a viable alternative treatment [173,180]. This finding is particularly relevant given the side effects associated with some conventional treatments, making caffeine an attractive option for patients seeking effective hair loss therapies with a potentially better safety profile. Moreover, the differential effects of caffeine on male and female hair follicles have been explored, revealing that caffeine can stimulate hair growth in both sexes, albeit through potentially different mechanisms or degrees of effectiveness [174,175]. This gender-specific response underscores the need for further research to fully understand how caffeine can be optimized for diverse populations experiencing hair loss.

Chlorogenic acids are another important group of compounds in *Coffea arabica* that contribute to hair growth. These compounds exhibit antioxidant and anti-inflammatory properties, which can help create a healthier environment for hair follicles. Chlorogenic acids have been shown to enhance blood circulation in the scalp, providing essential nutrients to hair follicles. Concentrations of 1% to 3% of chlorogenic acids in topical formulations have been reported to promote hair growth by improving follicle health and reducing inflammation [181].

Chlorogenic acids derived from *Coffea arabica* have garnered attention for their potential role in follicle stimulation and hair growth. These compounds are primarily recognized for their antioxidant properties, which are crucial in mitigating oxidative stress that can adversely affect hair follicles. The activation of the β-catenin signaling pathway is one of the mechanisms through which chlorogenic acids exert their effects. This pathway is vital for hair follicle development and cycling, as it promotes the transition from the telogen (resting) phase to the anagen (growth) phase of hair follicles. Research indicates that chlorogenic acid can prevent the phosphorylation of β-catenin, thereby inhibiting its degradation and facilitating its accumulation in the nucleus, where it can activate target genes essential for hair growth [28,182]. Additionally, chlorogenic acid has been shown to stimulate the expression of growth factors such as insulin-like growth factor 1 (IGF-1) and keratinocyte growth factor (KGF), both of which are critical for hair follicle proliferation and maintenance [28]. The presence of these growth factors is associated with enhanced hair follicle activity, suggesting that chlorogenic acids could play a significant role in promoting hair growth through these pathways. Moreover, the antioxidant properties of chlorogenic acids contribute to their hair growth-promoting effects. They scavenge free radicals and reduce oxidative damage, which can lead to hair follicle miniaturization and hair loss [183]. The high phenolic content in *Coffea arabica* extracts correlates with significant antioxidant activity, further supporting the notion that these extracts can protect hair follicles from oxidative stress [63]. This protective effect is essential not only for maintaining existing hair but also for encouraging the growth of new hair. Clinical applications of *Coffea arabica* extracts, particularly those enriched with chlorogenic acids, have shown promise in treating various forms of alopecia. Products containing these extracts have been reported to improve hair density and reduce hair loss in clinical trials, demonstrating their efficacy in real-world applications [167,168,184].

## 4. Applications of *Coffea arabica* Extracts in Dermato-Cosmetic Products

The applications of *Coffea arabica* in dermato-cosmetic products are expanding, driven by its phytochemical profile and beneficial properties for skin and hair health. The unique properties of natural products from *Coffea arabica*, including their antioxidant, anti-inflammatory, antimicrobial, and hair growth-promoting effects, make them a versatile ingredient in various cosmetic formulations.

The efficacy of *Coffea arabica* extracts in cosmetic formulations is linked to the method used for their obtainment. Most extraction techniques employ water or ethanol–water mixtures, yielding polar compounds like chlorogenic acid, phenolic acids, and caffeine [185]. These extracts can subsequently be used in creams and hydrogels. Common extraction techniques include the following:

- Water extraction: This method is effective for retaining water-soluble compounds such as chlorogenic acids and caffeine. Extraction by water generally employs boiling water, but subcritical water extraction has also been used [185].
- Extraction with mixtures of ethanol and water, going from 20% [186] to 70% ethanol [88,187]: Thus enables the obtainment of extracts that contain substantial amounts of polyphenols that are valued for the inclusion in dermato-cosmetic products. During a cold extraction method, Affonso and co-workers employed 70% (*v*/*v*) ethanol solution to extract the bioactive compounds from both green and roasted coffee seed press cake. The extract obtained from green coffee press cake was particularly rich in chlorogenic acid (11.11 mg/g), followed by caffeine (4.5 mg/g), trigonelline (1.55 mg/g), ferulic acid (1.40 mg/g), protocatechuic acid (1.20 mg/g), and phenolic acids. The extract obtained from roasted coffee press cake had, as a major ingredient, caffeine (5.6 mg/g), followed by lower amounts of chlorogenic acid (1.95 mg/g). Both extracts were incorporated, separately, into carpool-based hydrogels for topical application, in concentrations of 10%, and compared with hydrogels containing 3% chlorogenic acid (*w*/*v*) and 1% allantoin, respectively, in order to evaluate the cicatrizing effect. The extract from the green bean press cake showed the highest wound healing activity, followed by that of allantoin and roasted seed extract. Beneficial effects on cellular repair processes and reductions in oxidative stress were highlighted for the coffee extracts [88].
- Extraction with ethanol: Ethanol extracts can capture a broader range of phytochemicals, including polar and non-polar compounds. These extracts may be used for formulating creams and gels. For instance, Hadiningrat and co-workers prepared an ethanol extract from *Coffea arabica* leaves. Several concentrations of this extract (2.5%, 5%, and 7.5%, respectively) were tested after incorporation in a gel. The ethanol extract from the leaves of *Coffea arabica* inhibited, in a concentration-dependent manner, the increase in matrix metalloproteinase-1 (MMP-1) levels, an enzyme associated with collagen degradation and skin aging. Administration of the extract led to an increase in collagen density in the skin of Wistar rats exposed to UV-B light, suggesting a beneficial effect on skin health and the prevention of photoaging [188].
- Cold pressing: This technique is often used for extracting oils from both green and roasted *Coffea arabica* seeds, preserving essential fatty acids and antioxidants. The major secondary metabolites in cold-pressed oils from coffee seeds are the diterpenes kahweol and cafestol [118]. Coffee oils can be incorporated into cosmetic products in concentrations of up to 15%, significantly reducing transepidermal water loss [189].

In addition to topical applications, *Coffea arabica* has also been explored for its potential in oral supplements aimed at improving skin health from within. The intake of *Coffea arabica* extracts, particularly those rich in chlorogenic acids, has been associated with enhanced skin hydration and elasticity. A study indicated that oral supplementation with a *Coffea arabica* pulp drink at a volume of 50 mL per day resulted in significant improvements in skin moisture levels and overall skin appearance after eight weeks of continuous use [190]. This systemic approach complements topical applications, providing a holistic strategy for skin care.

*Coffea arabica* can be synergistically combined with other natural ingredients to enhance its effects [191]. These extracts can be utilized in various forms, such as oils, creams, and serums [4,5]. Furthermore, the incorporation of these extracts into topical formulations can enhance skin hydration and elasticity, thereby improving overall skin health [192]. For example, combining *Coffea arabica* extract with hyaluronic acid can improve skin hydration and elasticity. Formulations containing 2% *Coffea arabica* extract and 1% hyaluronic acid have shown promising results in clinical studies [167]. Furthermore, the use of *Coffea arabica* extracts in combination with other anti-aging agents, such as retinoids and peptides, can create synergistic effects that enhance overall efficacy [193].

In addition to traditional formulations, innovative delivery systems have been developed to enhance the bioavailability and efficacy of *Coffea arabica* extracts. Thus, encapsulation techniques can be used. Methods such as liposomes, niosomes, and microencapsulation can protect sensitive compounds from degradation and improve their penetration into the skin. Liposomes, which are spherical vesicles composed of phospholipids, can encapsulate hydrophilic and lipophilic substances, thereby protecting them from degradation and facilitating their release into the targeted skin layers. Saewan and co-workers encapsulated coffee berry extract in nanoliposomes and tested their effects in vitro (on human dermal fibroblasts) and in vivo (on human skin). They observed that the encapsulation of the extract enhanced its stability and increased its penetration through the skin while maintaining its anti-aging effects (improved superoxide-dismutase effect and lowered collagenase activity) [28]. The effects of liposomes prepared from aqueous and methanol coffee seed extracts with the aid of soy lecithin were also investigated. The obtained liposomes displayed a high antioxidant activity in several in vitro experiments [194]. Another innovative approach to utilizing *Coffea arabica* in dermatological applications is through the development of microemulsions. Microemulsions are thermodynamically stable systems that enhance the solubility and bioavailability of active ingredients. By formulating *Coffea arabica* extracts into microemulsions, researchers have demonstrated improved skin penetration and efficacy of the bioactive compounds. For instance, a microemulsion containing *Coffea arabica* extract was shown to enhance the delivery of antioxidants to the skin, resulting in improved skin hydration and elasticity [195].

Nanotechnology has also emerged as a promising approach to enhance the properties of coffee extracts. For example, the use of reduced graphene oxide (rGO) nanocomposites combined with *Coffea arabica* extracts has been explored for their anti-inflammatory and antioxidant properties. This method not only improves the stability of the active compounds but also facilitates their penetration into the deep layers of the skin, thus enhancing their therapeutic effects. The synthesis of rGO using *Coffea arabica* leaf extract exemplifies an environmentally friendly approach for the development of advanced delivery systems that can be used in cosmetic applications [196].

The pH of cosmetic formulations can significantly affect the stability and activity of *Coffea arabica* extracts. Maintaining a slightly acid pH (around 5.5) is crucial not only for skin-friendly products but also for preserving the integrity of chlorogenic acids and other active compounds [197]. Formulations should be tested for pH stability to ensure optimal performance [198].

*Coffea arabica* extracts can be incorporated into creams and gels designed for specific skin concerns, such as anti-aging and hydration. Formulations containing 2.5% extract from *Coffea arabica* silverskin have been shown to improve skin texture and reduce the appearance of fine lines [126]. Additionally, *Coffea arabica* has been incorporated into anti-aging creams and serums. The seed oil of *Coffea arabica* is rich in antioxidants, particularly chlorogenic acids, which have been shown to improve collagen and elastin production in skin fibroblasts. This property is particularly beneficial for combating signs of aging, such as wrinkles and loss of skin elasticity. A typical formulation may include *Coffea arabica* seed oil, combined with other emollients and humectants to enhance skin hydration and texture [126,199].

Lightweight serums with high concentrations of *Coffea arabica* can provide targeted treatment for skin issues such as hyperpigmentation and inflammation. These serums can be formulated with additional antioxidants and peptides to enhance their efficacy [200]. One of the primary formulations utilizing *Coffea arabica* is the sunscreen gel preparation derived from its leaf extract. Research indicates that the ethanol extract of *Coffea arabica* leaves contains phenolic and flavonoid compounds that exhibit significant UV absorption properties, making them suitable for sunscreen applications. A study demonstrated that a gel formulation containing 70% ethanol extract of *Coffea arabica* leaves could effectively shield the skin from harmful UV radiation, showing promising sun protection factor (SPF) results. The higher concentrations of the extract in the gel formulation were correlated with increased SPF values, thereby enhancing the protective efficacy against UV-induced skin damage [112].

The versatility of *Coffea arabica* extends to its use in hair care products as well. *Coffea arabica* extracts can be included in shampoos, conditioners, and scalp treatments aimed at promoting hair growth and health. Formulations containing 3% to 5% *Coffea arabica* extract have been shown to stimulate hair follicles and improve hair density [167,201]. The antioxidant and anti-inflammatory properties of its extracts can be beneficial for scalp health and hair growth. Formulations such as shampoos and conditioners incorporating *Coffea arabica* extracts at concentrations of 1% to 3% have been developed, targeting issues such as dandruff and hair thinning. The application of these formulations has been linked to improved scalp condition and enhanced hair vitality, attributed to the nourishing effects of the bioactive compounds present in *Coffea arabica* [145].

Advanced delivery systems such as microneedles and iontophoresis can enhance the penetration of a wide range of cosmetic actives into deeper skin layers. These technologies can be particularly beneficial for delivering active compounds including caffeine in skin rejuvenation and anticellulite treatments [202].

The sustainability aspect of using *Coffea arabica* in cosmetic formulations is also noteworthy. With increasing awareness of environmental issues, the cosmetic industry is shifting towards utilizing by-products from coffee production, such as coffee silverskin and spent coffee grounds [37,126]. These by-products, rich in antioxidants and other beneficial compounds, can be incorporated into formulations, promoting sustainability while harnessing the beneficial properties of *Coffea arabica*. For instance, formulations utilizing coffee silverskin have shown promise in enhancing skin barrier function and providing antioxidant protection [37,145,192,203].

*Coffea arabica* offers a wealth of applications in dermato-cosmetic products, supported by its rich phytochemical profile and beneficial properties. Effective formulation strategies, optimal extraction methods, ingredient combinations, and advanced delivery systems can enhance the efficacy of *Coffea arabica* extracts in skin care and hair care products. From sunscreen formulations to anti-aging creams, microemulsions, shampoos, and oral supplements, the versatility of *Coffea arabica* is evident in its ability to address various skin concerns. The ongoing research into its bioactive compounds and their effects on skin health will undoubtedly lead to the development of products that cater to the diverse needs of consumers. As research continues to unveil the potential of this remarkable plant, the integration of *Coffea arabica* extracts and compounds into improved delivery systems will likely expand, offering consumers effective and sustainable options for skin care.

### Safety Issues with Regard to Applications on the Skin

Although the safety of topical formulations is an important aspect, the scientific literature shows a reduced number of studies in this regard. The safety of coffee silverskin extracts was assessed by an in vitro model of skin (EpiSkinTM), a model of human cornea epithelium (SkinEthicsTMHCE) as well as in vivo [149]. The subjects of the research were represented by three extracts in order to evaluate different compound fractions: water, 50% ethanol, and ethanol were used for extraction, respectively. The content of 5-hydroxymethyl furfural, an irritant and cytotoxic compound that is formed during coffee roasting, was monitored by HPLC in all extracts, along with caffeine concentration. Of all extracts, the hydro-alcoholic one was best tolerated, inducing no morphologic issues of the skin and ocular models and inducing the lowest interleukin-1α release. However, all the extracts proved to be safe and produced no statistically significant irritation [149].

Wagemaker and co-workers evaluated the safety of the unsaponifiable fraction from green coffee oil, known to contain photoprotective compounds. The safety was assessed with regard to the effect on brine shrimp viability and cytotoxicity test on keratinocytes. The tests highlighted significant cytotoxicity in both assays, prompting attention to the use of unsaponifiable coffee oil in cosmetic preparations despite the high UV-protective potential [204]. However, the investigation of green coffee oil, either pure or in cosmetic formulations of up to 15%, showed no cytotoxic effects. Furthermore, its use on volunteers for a duration of three days induced no erythema [205].

The safety of a cream containing coffee pulp extracts was studied in a rodent animal model. Three extract concentrations were investigated: 5%, 7.5%, and 10%. The use of the extracts was considered safe as no irritation occurred. Furthermore, rats exposed to UV light and treated with coffee pulp extract showed lower erythema and wrinkles [206].

## 5. Conclusions

The phytochemical analysis of *Coffea arabica* reveals a rich profile of bioactive compounds, including caffeine, chlorogenic acids, and various diterpenes, which contribute to its antioxidant, anti-inflammatory, and antimicrobial properties. Notably, the antioxidant capacity of *Coffea arabica* extracts has been well documented, with studies indicating significant radical scavenging activity, which can be attributed to its high phenolic content. This antioxidant activity is crucial for protecting skin cells from oxidative stress, a key factor in skin aging and various dermatological conditions.

Furthermore, the dermato-cosmetic effects of *Coffea arabica* extend beyond antioxidant properties. The extract has demonstrated potential in anticellulite and anti-aging applications, as well as in promoting skin hydration and providing UV protection. The emollient properties of *Coffea arabica* also make it a valuable ingredient for enhancing skin moisture retention, thereby improving overall skin texture and appearance. Additionally, its antimicrobial properties suggest a role in preventing skin infections and promoting scalp health, which could stimulate hair growth.

Despite these promising findings, there are several research gaps that need to be addressed to fully harness the potential of *Coffea arabica* in dermato-cosmetic applications. Future research should focus on the standardization of extraction methods to optimize the bioavailability and stability of active compounds. Moreover, clinical studies are necessary to validate the efficacy of *Coffea arabica* in various dermato-cosmetic formulations and to understand its long-term effects on skin health. Investigating the synergistic effects of *Coffea arabica* with other ingredients could also enhance its therapeutic potential and broaden its applications in skin care products. Further investigations into the safety of *Coffea arabica* extracts are also warranted.

Overall, *Coffea arabica* presents a multifaceted opportunity as a component of dermato-cosmetic formulations. Its diverse phytochemical profile and proven bioactive properties make it a valuable ingredient for enhancing skin health and appearance. Continued research and development will be essential to unlock its full potential and address existing gaps in knowledge regarding its applications in the cosmetic industry.

## Figures and Tables

**Table 1:** Chemical structures and structural diversity of key secondary metabolites from *Coffea arabica*. The chemical structures were created using KingDrawHD v1.4.5.-20230617 software.

Secondary Metabolite Class	Key Metabolites	Chemical Structures	Structural Diversity
Alkaloids	Caffeine	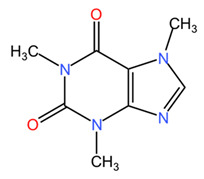	Functional Groups: Alkaloid (purine type), amine. Structural Particularities: Contains a xanthine skeleton with three methyl groups (N-3, N-7, N-1).
	Trigonelline	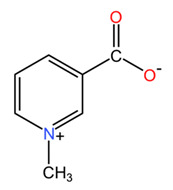	Functional Groups: Alkaloid, carboxylic acid, amine. Structural Particularities: Contains a pyridine ring with a carboxyl group, derived from nicotinic acid.
Phenolic acids	Chlorogenic acid	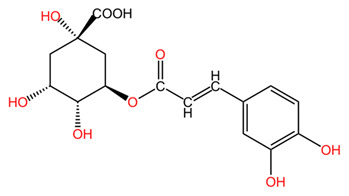	Functional Groups: Ester, phenolic acid. Structural Particularities: Contains a caffeic acid and quinic acid moiety connected by an ester bond.
	Caffeic acid	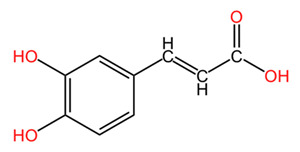	Functional Groups: Phenolic acid. Structural Particularities: Features a trans double bond in the side chain, enhancing antioxidant properties.
Flavonoids	Quercetin	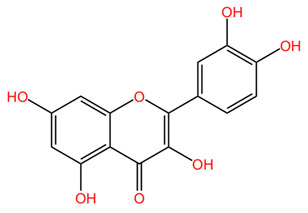	Functional Groups: Flavonoid (phenolic group). Structural Particularities: Contains multiple hydroxyl groups and a keto group, allowing for extensive hydrogen bonding.
	Kaempferol	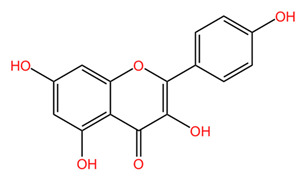	Functional Groups: Flavonoid (phenolic group). Structural Particularities: Similar to quercetin but lacks one hydroxyl group.
	Rutin	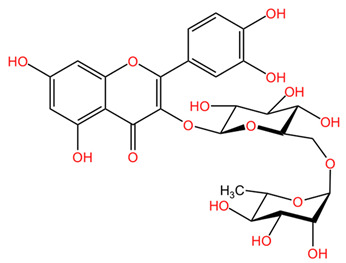	Functional Groups: Flavonoid (glycoside, phenolic). Structural Particularities: A glycoside of quercetin; contains a disaccharide (rutinose) attached, increasing solubility.
Xanthones	Mangiferin	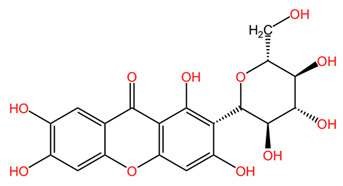	Functional Groups: Xanthone, phenolic. Structural Particularities: Comprising of a xanthone core with a sugar moiety; exhibits high antioxidant activity.
Diterpenes	Cafestol	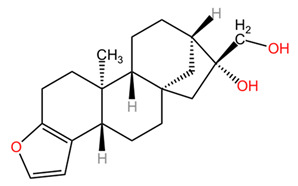	Functional Groups: Diterpene, alcohol. Structural Particularities: Contains a bicyclic structure with multiple hydroxyl (-OH) functional groups.
	Kahweol	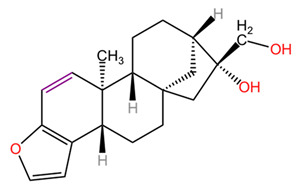	Functional Groups: Diterpene, alcohol. Structural Particularities: Similar to cafestol but with an additional methylene group, influencing its biological activity.

**Table 2:** Comparative concentrations of the main representative secondary metabolites in the leaves, green seeds, and roasted seeds of *Coffea arabica* [24].

Metabolite	Leaves	Green Seeds	Roasted Seeds
Caffeine	2.5–6.0	7.6–29.0	5.3–20.3
Trigonelline	<3.0	8.8–27.6	<2.5
Theobromine	<0.1	<0.1	<0.1
Theophylline	<0.1	<0.1	<0.1
Chlorogenic acids	19.2–39.6	57.0–80.3	<3.0
Mangiferin	0.5–9.2	ND	ND
Cafestol	<6.0	2.7–11.0	<5.0
Kahweol	ND	1.1–6.7	<10.0

Concentrations are expressed as mg/g dry matter. Chlorogenic acids are expressed as mg of total chlorogenic acids/g of dry matter; ND = not detected.

**Table 3:** Comparative concentrations of the main representative primary metabolites in the leaves, green seeds, and roasted seeds of *Coffea arabica*.

Metabolite	Leaves	Green Seeds	Roasted Seeds
Carbohydrates	51.0–63.9% [46]	59–61% [57]	38–42% [57]
Lipids	5–10% [74]	11–17% [57]	11–17% [57]
Proteins	5–8% [74]	10–16% [57]	8–14% [57]

Concentrations are expressed as % of dry matter.

## Data Availability

There are no additional data to be published.
